# Genetic Factors and Host Traits Predict Spore Morphology for a Butterfly Pathogen

**DOI:** 10.3390/insects4030447

**Published:** 2013-08-28

**Authors:** Sarah E. Sander, Sonia Altizer, Jacobus C. de Roode, Andrew K. Davis

**Affiliations:** 1 Odum School of Ecology, University of Georgia, Athens, GA 30602, USA; E-Mail: sesanderuga@gmail.com (S.E.S.); saltizer@uga.edu (S.A.); 2 Department of Biology, Emory University, Atlanta, GA 30322, USA; E-Mail: jacobus.de.roode@gmail.com

**Keywords:** monarch butterfly, *Danaus plexippus*, neogregarine, *Ophryocystis elektroscirrha*, aspect ratio, spore size, parasite load

## Abstract

Monarch butterflies (*Danaus plexippus*) throughout the world are commonly infected by the specialist pathogen *Ophryocystis elektroscirrha* (*OE*). This protozoan is transmitted when larvae ingest infectious stages (spores) scattered onto host plant leaves by infected adults. Parasites replicate internally during larval and pupal stages, and adult monarchs emerge covered with millions of dormant spores on the outsides of their bodies. Across multiple monarch populations, *OE* varies in prevalence and virulence. Here, we examined geographic and genetic variation in *OE* spore morphology using clonal parasite lineages derived from each of four host populations (eastern and western North America, South Florida and Hawaii). Spores were harvested from experimentally inoculated, captive-reared adult monarchs. Using light microscopy and digital image analysis, we measured the size, shape and color of 30 replicate spores per host. Analyses examined predictors of spore morphology, including parasite source population and clone, parasite load, and the following host traits: family line, sex, wing area, and wing color (orange and black pigmentation). Results showed significant differences in spore size and shape among parasite clones, suggesting genetic determinants of morphological variation. Spore size also increased with monarch wing size, and monarchs with larger and darker orange wings tended to have darker colored spores, consistent with the idea that parasite development depends on variation in host quality and resources. We found no evidence for effects of source population on variation in spore morphology. Collectively, these results provide support for heritable variation in spore morphology and a role for host traits in affecting parasite development.

## 1. Introduction

Many parasites produce dormant transmission stages such as eggs or spores that persist outside of the host before encountering and infecting a new host [1]. In some cases, these stages survive for only hours outside of their host, whereas other parasites produce thick-walled and longer-lived spores. Because parasite fitness depends on successful transmission, it is likely that the morphology of these dormant stages has been shaped by selection for dispersal propensity, and also based on the ability of spores to withstand exposure to environmental stressors (e.g., desiccation, extreme temperatures, sunlight). For example, shape is known to affect the passive dispersal of fungal spores, with the optimal shape being an oval spore with tapered ends [2,3]. Additionally, melanism (dark coloration) has been associated with resistance to UV-damage in many systems including pathogenic bacteria and fungi [4,5,6]. Importantly, variation in environmental conditions and opportunities for transmission could produce geographic variation in the morphology of transmission stages and might support heritable variation for those traits within populations. In addition, because parasites rely on host-derived resources for their own replication and development, parasite traits such as size and pigmentation could further depend on host traits, especially those related to energy acquisition and allocation, and on total parasite load [7,8,9,10].

**Figure 1:**
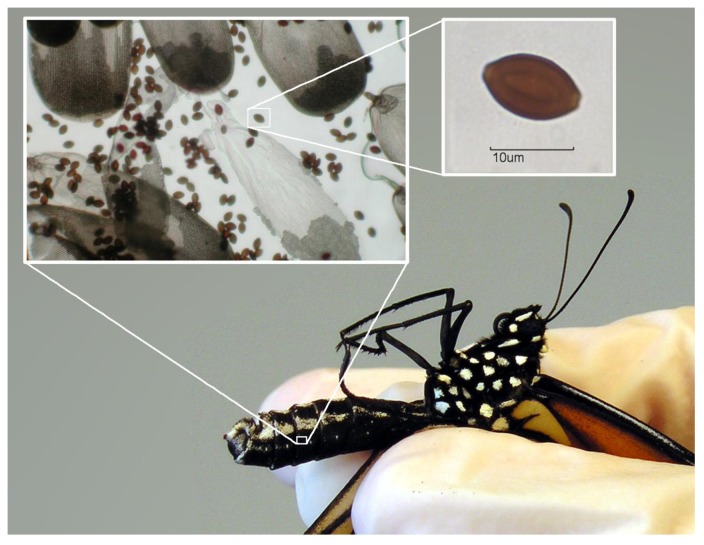
*Ophryocystis elektroscirrha* (*OE*) infection of a monarch butterfly. Adult monarchs emerge covered with millions of dormant, lemon-shaped, amber-walled spores (inset figure) on their abdomen. Small inset figure: A single spore showing correct 2D orientation to the field of view for morphometric analysis.

In this study, we examined the degree to which genetic and environmental factors, including host characteristics, predict morphological variation in a spore-producing insect pathogen. We focused on a neogregarine sporozoan *Ophryocystis elektroscirrha* (hereafter called *OE*; Figure 1) that commonly infects monarch butterflies, *Danaus plexippus*. This parasite produces external dormant transmission stages that are scattered by infected adults onto eggs and host plant surfaces [11,12]. Spores might persist in the environment for variable lengths of time and must be ingested by a larva to cause a new infection. *OE* can be transferred vertically, from infected adults to their progeny, and horizontally, when larvae ingest spores scattered onto host plants by unrelated adults [12,13]. Sporozoites (motile infective cells) are released in the larval gut, migrate to the hypoderm and undergo multiple phases of vegetative schizogony, whereby each parent cell can produce several hundred daughter cells [11]. Spore production occurs several days prior to adult eclosion, and infected adults emerge covered with dormant spores on the outside of their bodies, with the highest densities on their abdomens [14]. Parasites do not continue to replicate on adults, spores must be eaten by a larva to cause a new infection, and larva-to-larva transmission does not occur [15].

Infections by *OE* can be debilitating and result in reduced body size (which is often indexed by wing size, [16]), shorter adult lifespans, lower mating success, reduced flight performance and reduced black pigmentation on the wings [16,17,18,19]. The magnitude of these effects depends on the severity of infection [20], which can be indexed by counting the number of *OE* spores on the adult body. Infections are naturally present in monarch populations around the world; migratory populations have the lowest *OE* prevalence, and non-migratory populations often show very high prevalence of infection [21]. Past work has shown a strong genetic basis for the high levels of within-population variation in monarch susceptibility to infection and in *OE* virulence [22,23]. Moreover, parasite isolates from the longest-distance migratory population in eastern North America are less virulent than isolates from short-distance and non-migratory populations [20,22,24], suggesting differential selection on parasite traits across wild populations.

Our first goal was to ask whether parasite genotype and source population predict variation in morphological characteristics of *OE* spores using clonal parasite lineages isolated from four wild monarch populations. Although determining the function of *OE* spore traits is beyond the scope of this study, quantifying variation in these traits and establishing their underlying genetic basis is an important first step towards exploring their adaptive significance. A second goal of this study was to determine if variations in spore morphology are associated with host traits, including wing size and pigmentation. For example, larger spore size might correlate negatively with monarch wing size and/or wing darkness if parasite genotypes that produce larger spores also consume more host resources. Conversely, there is evidence from other parasite systems that host and parasite body size can be positively associated [7,8,9,10]. This is thought to be caused by larger hosts having more abundant internal resources, allowing for greater parasite growth, or it may simply be because larger hosts offer more physical space for parasite growth [7]. Most prior work on this trend focused on larger-bodied macroparasites, and it is unclear if this pattern can be extended to unicellular parasites like protozoa. A third goal was to examine whether spore morphology depends on the abundance or density of parasites within individual hosts. Experimental infections show high per capita replication rates of *OE* (between 10^4^ and 10^6^ parasites per ingested spore [18], and host resources available for parasite growth or pigmentation might become limiting at high parasite densities. Therefore, greater parasite replication could trade-off against spore size or other characteristics, leading us to predict a negative relationship between *OE* spore loads and measures of spore size and pigmentation.

## 2. Methods

*Host and parasite sources.* Wild-caught infected monarchs from each of 4 populations, Hawaii, South Florida, western North America, and eastern North America, were used to create a series of parasite clones (Table 1). These populations have been previously demonstrated to show sharply different levels of *OE* prevalence and, in some cases, virulence [21,24,25]. Although a high degree of genetic mixing is known to occur between monarchs across North America [26], other work showed evolutionary divergence in wing morphology among monarchs from the same populations examined here [27], suggesting the potential for evolutionary divergence in parasite traits.

Five clonal parasite lineages per source population were created by infecting caterpillars with single haploid parasite spores (as described in [18,22]), to produce a total of 20 parasite clonal lines. Butterfly lineages for experimental infections were derived from approximately 30 adult monarchs (spring migrants returning from Mexico) collected near San Antonio, Texas in April 2009. Laboratory-reared F1 offspring of these adults were fed greenhouse-raised cuttings of *Asclepias incarnata* according to protocols described in de Roode and Altizer [22]. Newly emerged uninfected adults were used to produce 3 non-inbred F2 family lines (hereafter referred to as host lineages).

To obtain parasites for morphometric analyses, spores from each clonal line were used to inoculate 3 replicate monarch larvae from each host lineage (for a total of 180 infected monarchs) with a dose of approximately 50 spores per individual. Inoculation methods followed previously developed protocols [22]. Larvae were housed individually in 1L plastic containers and reared to adulthood using cuttings of greenhouse-grown milkweed (*Asclepias incarnata*). Following eclosion, adults were stored in individual glassine envelopes at −20 °C for 6 months prior to morphometric analysis of their spores. A total of 164 monarchs survived to adulthood. A number of specimens were held for later experiments and were not measured here, resulting in 126 infected adult monarchs for which complete data were available (Table 1), with at least 5 individuals per parasite clone.

*Digital imaging and morphometric assays.* To isolate individual spores, we wiped a cotton swab along each butterfly’s abdomen and tapped the swab on a glass slide to deposit parasite spores. Spores were fixed to the slide using transparent tape and viewed with a light microscope under 1,000 × oil immersion. Photographs were taken of single spores that were oriented to present a full 2D view to the camera. Spore images were measured in Adobe Photoshop (Adobe Systems, Mountain View, CA, USA) with the Fovea Pro image analysis plug-in (Reindeer Graphics, Inc., Asheville, NC, USA). We recorded five measures for each spore: (1) two-dimensional area, (2) length of the longest axis, (3) breadth of the longest perpendicular axis, (4) aspect ratio (length divided by breadth), and (5) spore darkness. Darkness was measured as the average pixel density within the spore image; density is measured on a 0–255 scale with lower numbers representing darker shades [19,28]. We considered spore area, aspect ratio and darkness to be the best variables for analyses of spore size, shape and color, respectively. A total of 30 spores were measured per individual monarch; from these, we obtained an average value of each spore measure for each parasite clone by individual host combination.

**Table 1:** Parasite sources, based on sites and dates of capture of infected butterflies, and number of infected monarchs from each clonal line of *OE* used in this study, including only hosts for which wing data were available (last column, n = 126 total).

Monarch Population	Capture Site *	Capture Date *	Clone ID	# Monarchs per Clone
**Western N. America**	Santa Barbara, CA	Apr-05	C2	8
	Pismo Beach, CA	May-03	C8	7
	Pismo Beach, CA	Feb-05	C10	5
	Santa Cruz, CA	Mar-05	C14	6
	Big Sur, CA	Mar-05	C16	5
**Eastern N. America**	Cape May, NJ	Oct-01	E3	5
	Charlottesville, VA	Sep-04	E6	6
	St. Paul, MN	Jul-05	E10	6
	Sweet Briar, VA	Jul-05	E12	5
	Sweet Briar, VA	Jul-05	E13	7
**Hawaii**	Oahu, HI	Feb-03	H1	8
	Big Island, HI	Feb-03	H3	7
	Big Island, HI	Feb-03	H5	6
	Big Island, HI	Feb-03	H7	6
	Big Island, HI	Feb-03	H10	5
**South Florida**	Miami, FL	Jan-04	F3	7
	Miami, FL	May-04	F11	8
	Miami, FL	May-04	F12	7
	Miami, FL	Jan-04	F14	6
	Miami, FL	Apr-04	F16	6

* Capture site and date of original infected monarchs used to create clonal lines.

**Figure 2:**
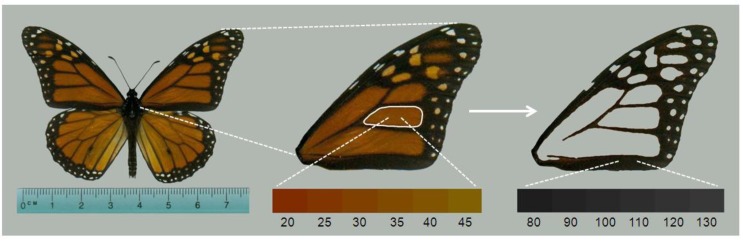
Wing parameters measured on monarchs. One forewing was removed from the specimen and scanned. Image analysis produced measures of forewing area, hue score of the orange color in the central cell (white outline), and melanism score (measured with pixel density) of all black areas of forewing. Lower bars (connected to images by white dashed lines) show the range of hue and melanism scores typically seen in monarch butterflies and in this study.

We next used image analysis to quantify adult monarch morphometric data that correspond to known effects of *OE* on monarch fitness. We scanned the left forewing of each monarch (Figure 2) using a flatbed scanner at a resolution of 300 dpi. We ensured that there was no color-correction by the scanner software, and that all settings were consistent across images. If the left wing was damaged during storage or spore collection, the right wing was used. From the forewing images, we measured the total forewing area (mm^2^) to serve as an index of butterfly size. Wing area predicts mating success in males [29] and adult longevity in captive monarchs [30], and is generally reduced by *OE* infection [18]. We also used a built-in routine to measure the hue of the orange color in the center wing cell (Figure 2). This measure is the average of all pixel hue values in the selected region, and is measured in degrees (0–360). Most monarchs have hue scores between 20 and 45 (Figure 2), with lower scores indicating darker orange shades (nearing red) and higher scores indicating more yellowed shades of orange. 

Redder hues in monarchs correspond to higher flight performance [31] and greater longevity in captive monarchs [30]. Finally, we measured the melanism (*i.e*. degree of black) of monarch wings by selecting all black portions of the forewing to obtain the average pixel density score (Figure 2). This is a value from 0–255 with lower numbers representing darker black pigmentation [19,28,32].

*Spore load.* We estimated the total number of spores on adult monarchs as described in de Roode *et al*. [18]. Briefly, the abdomen was removed from each butterfly and vortexed at high speed for 5 minutes in 5 mL of deionized water. Spores were counted using 12 replicate 0.1 mm^3^ chambers from a hemocytometer slide. This provides an estimate of parasite replication within hosts inoculated with known parasite doses [18]. Log_10_-transformed spore load was included as a covariate in analyses of predictors of spore morphology.

**Figure 3:**
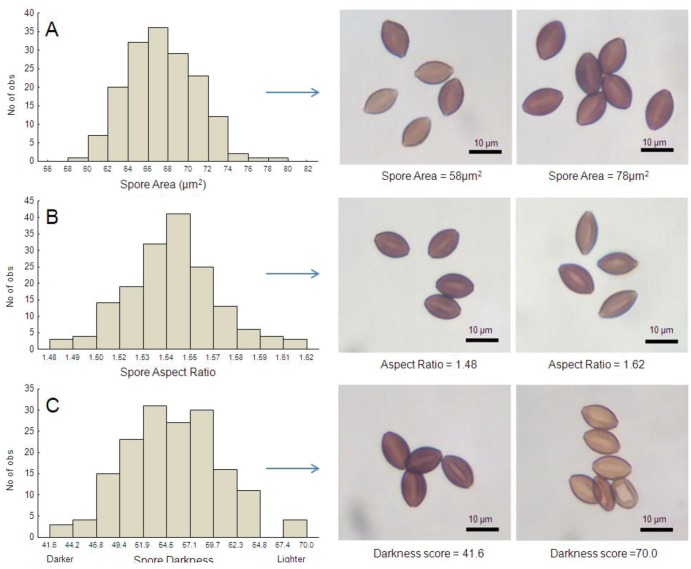
Frequency distribution of *OE* spore morphometric traits measured in this study, including spore size (2-dimensional area, **A**), shape (aspect ratio [length/width], **B**) and color (darkness, **C**). Images to the right of each graph are photomicrographs of spores that represent the upper and lower range of measurements. Values used to generate histograms are the means per host individual (averaged across 30 spores per monarch).

*Data Analysis.* Analyses of spore characteristics were based on averages at the level of individual monarch, where each monarch captured a specific host by parasite genotype combination. Variation in spore measures within individual monarchs was low (*i.e.*, the within-monarch standard error was less than 3% of the mean trait value for all traits examined here). With butterfly as the unit of observation, all three spore morphology variables (area, aspect ratio and darkness) were normally-distributed (Figure 3). We first used Pearson correlation tests to examine relationships between all three spore morphology variables. We next used general linear models to examine relationships between spore variables (tested independently) and all potential explanatory variables including population origin, *OE* clone (nested within source population), host genetic lineage and host sex. We also included several continuous covariates including log_10_ spore load, forewing size (area), forewing orange color (hue) and melanism (Full model: Spore trait = Population + Clone(Population) + Host Lineage + Host sex + Log_10_Spore load + Wing Size + Wing Hue + Wing Melanism). Parasite clone and host lineage were treated as random effects. Interaction effects were not included owing to limitations on sample size and model degrees of freedom. All analyses were conducted using the Statistica 6.1 software package [33].

## 3. Results

All three spore trait measures examined here showed substantial variation among butterfly hosts and parasite clones: The mean (two-dimensional) surface area of *OE* spores ranged from 58.15 mm^2^ to 78.02 mm^2^ (Figure 3A). The mean spore aspect ratio per butterfly varied from 1.48 to 1.62 (Figure 3B), meaning that some spores were more elongated than others, and we also observed variation in spore darkness (Figure 3C). Spore area correlated negatively with spore darkness among individual hosts (r = −0.426, N = 164, *p* < 0.001; here lower numbers represent darker shades), indicating that larger spores tended to be darker in color. We observed no significant relationship between spore area and spore aspect ratio (r = 0.172, *p* = 0.054), or between spore aspect ratio and spore color (r = 0.152, *p* = 0.089).

**Figure 4:**
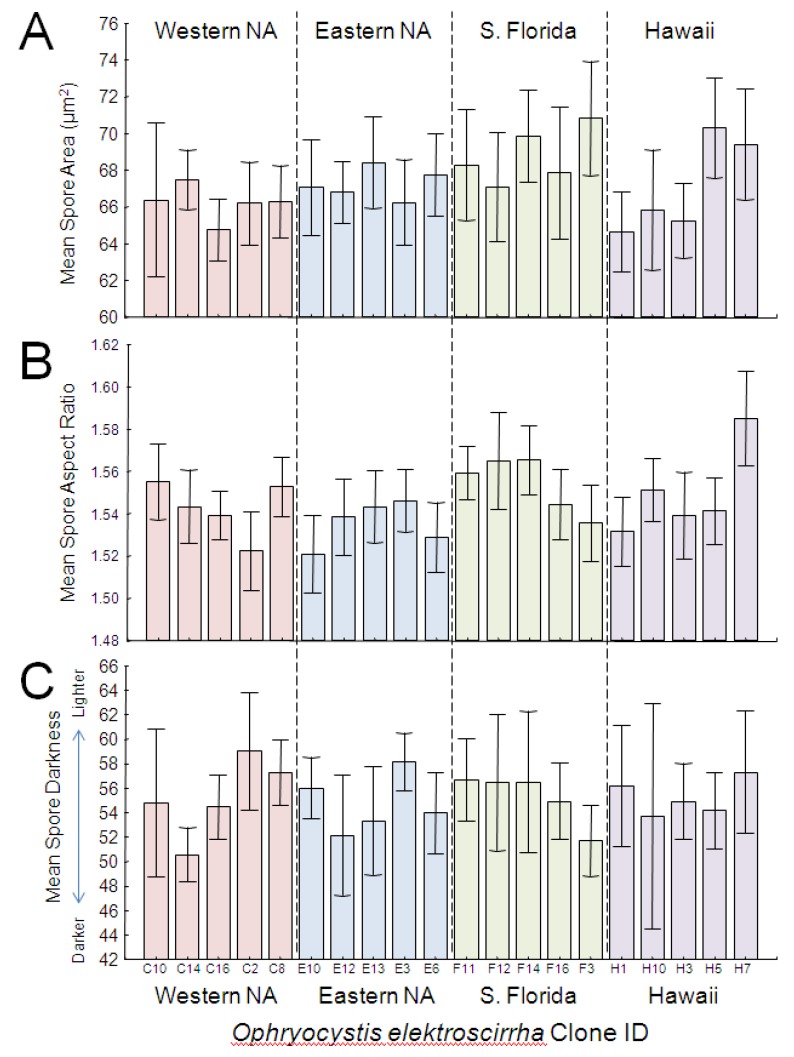
Average spore area (**A**), aspect ratio (**B**) and darkness score (**C**) of *OE* spores across monarch clones, arranged numerically within each source population. Eastern and western NA are from migratory monarch populations within North America. S. Florida refers to parasites obtained from a year-round resident (non-migratory) population near Miami, FL, USA, and Hawaii refers to parasites from non-migratory monarchs that inhabit the Hawaiian islands. Error bars represent 95% CI.

**Figure 5:**
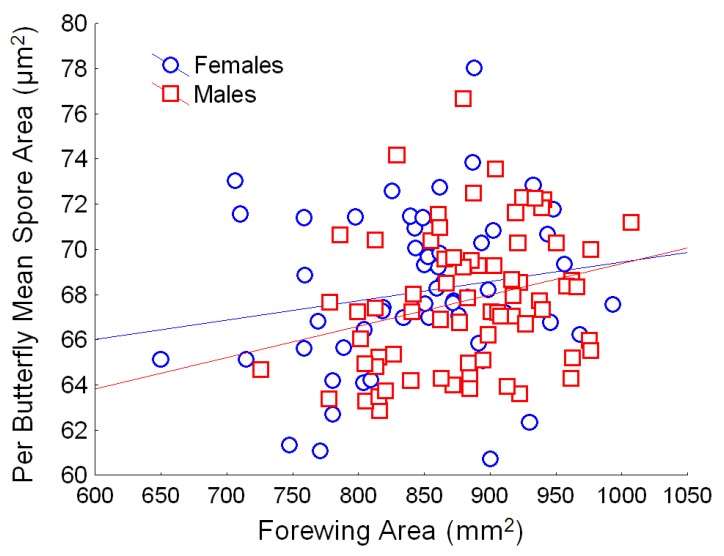
Relationship between butterfly size (forewing area, mm^2^) and *OE* spore area (mean per butterfly, in µm^2^) for males (red) and females (blue). Simple regression relationship (excluding other predictors from the full model) for females: Y = 60.9 + 0.010X; r^2^ = 0.032; for males: Y = 55.5 + 0.013X; r^2^ = 0.070.

GLM analysis showed that parasite clone explained a significant proportion of the variation in spore area (Table 2; Figure 4A). Both large and small clones were represented in each source population; spores from South Florida were largest and spores from western North America were smallest, but the overall effect of population origin was not significant. Effect size estimates showed that clone differences accounted for the greatest proportion (over 25%) of the variation in spore area. Host genetic lineage and sex further predicted differences in spore area (Table 2); in particular, females had slightly larger spores (*x* = 67.7 µm^2^, SD = 3.6) than males (*x* = 67.3 µm^2^, SD = 3.3). Spore area also increased significantly with butterfly wing area (Table 2), and this trend was evident for both male and female monarchs (Figure 5).

**Table 2:** Summary of general linear models examining predictors of spore morphology (area, aspect ratio and darkness). Significant predictor variables are highlighted. Analyses were based on averages among spores at the level of individual butterfly (n = 126). Estimated effect sizes for significant predictor variables are indicated by partial Eta squared values (n_p_^2^).

Response	Predictor	Df	MS	F	p	n_p_^2^
****Spore Area****	**Host Lineage**	**2**	**23.44**	**3.27**	**0.042**	**0.037**
	**Sex**	**1**	**33.36**	**4.66**	**0.033**	**0.026**
	***OE* Clone(Population)**	**16**	**20.41**	**2.85**	**0.001**	**0.256**
	Population	3	32.29	1.65 *	0.216	–
	**Wing Area**	**1**	**82.50**	**11.52**	**0.001**	**0.064**
	Orange Hue	1	13.33	1.86	0.176	–
	Wing Melanism	1	0.65	0.092	0.763	–
	Log_10_Spore load	1	0.10	0.014	0.907	–
	Error	98	7.16			
****Spore Aspect Ratio****	Host Lineage	2	0.0002	0.377	0.687	–
	Sex	1	0.0001	0.252	0.617	–
	***OE* Clone(Population)**	**16**	**0.0014**	**3.218**	**0.000**	**0.284**
	Population	3	0.0012	0.905 *	0.460	–
	Wing Area	1	0.0011	2.440	0.121	–
	Orange Hue	1	0.0001	0.320	0.573	–
	Wing Melanism	1	0.0005	1.131	0.290	–
	Log_10_Spore load	1	0.0004	0.929	0.337	–
	Error	98	0.0004			
****Spore Darkness****	**Host Lineage**	**2**	**88.75**	**3.78**	**0.026**	**0.048**
	**Sex**	**1**	**107.73**	**4.59**	**0.035**	**0.029**
	*OE* Clone(Population)	16	36.25	1.54	0.100	–
	Population	3	2.39	0.067 *	0.976	–
	**Wing Area**	**1**	**136.54**	**5.82**	**0.018**	**0.037**
	**Orange Hue**	**1**	**243.53**	**10.37**	**0.002**	**0.065**
	Wing Melanism	1	22.86	0.97	0.326	–
	**Log_10_Spore load**	**1**	**192.44**	**8.20**	**0.005**	**0.051**
	Error	98	23.47			

* F-statistic for population effect used clone (population) MS as error term in denominator. All other tests use mean square error as denominator for F-tests.

Analysis showed a significant effect of clone, but not population origin, on spore aspect ratio (Table 2; Figure 4B). As with spore area, both elongated and rounder clones were observed within each source population (Figure 4B), and effect size estimates showed that clone differences accounted for the greatest proportion (over 28%) of the variation in spore aspect ratio. No other independent variables tested here predicted variation in spore aspect ratio (Table 2).

**Figure 6:**
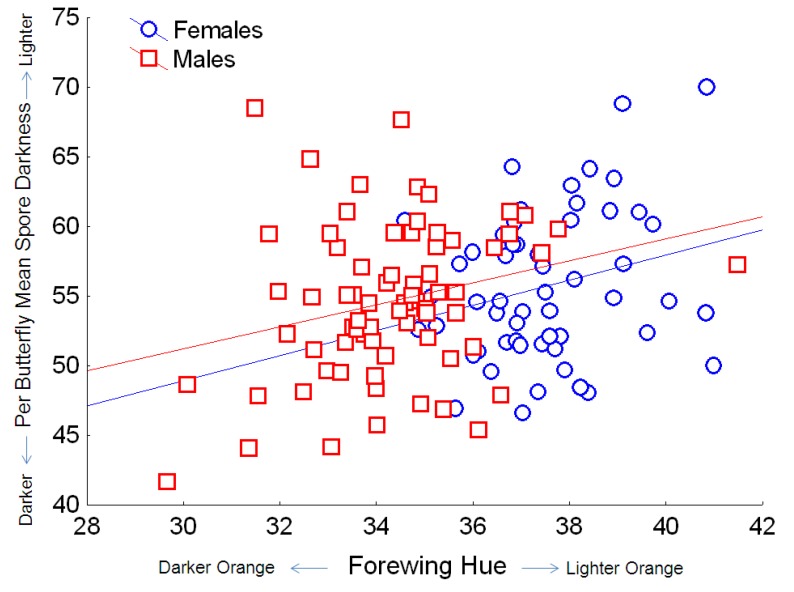
Relationship between the hue of orange on monarch wings (where lower values indicate redder hue, and higher values indicate yellow hue) and *OE* spore darkness (mean per butterfly, based on pixel density, for which the numerical score varies inversely with dark coloration). Simple regression relationship (excluding other predictors from the full model) for females: Y = 21.8 + 0.90X; r^2^ = 0.063; for males: Y = 27.5 + 0.79X; r^2^ = 0.070.

**Figure 7:**
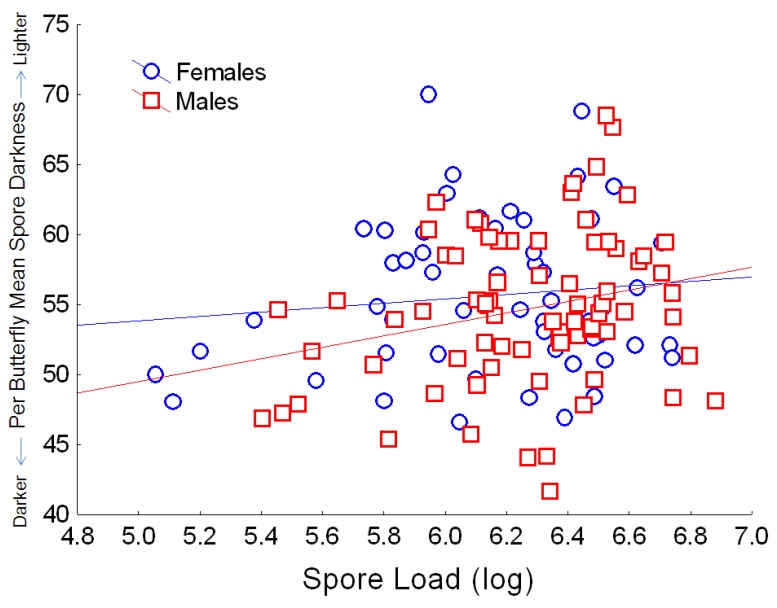
Relationship between the number of spores per butterfly (log_10_ spore load) and average spore darkness (mean per butterfly, based on pixel density, for which the numerical score varies inversely with dark coloration). Simple regression relationship (excluding other predictors from the full model) for females: Y = 46.0 + 1.56X; r^2^ = 0.013; for males: Y = 29.07 + 4.09X; r^2^ = 0.062.

Spore darkness depended only on host variables, and showed no association with source population or parasite clone. Both host lineage and sex were significantly associated with spore darkness (Table 2), with males tending to have darker spores than females. Monarch wing area and orange hue also predicted variation in spore darkness (Table 2). Monarchs with larger wings had darker spores, and this effect was consistent for both males and females. Monarchs with darker orange (*i.e*., redder) wings had darker *OE* spores (Figure 6), and this effect was similar for both males and females, which naturally differ in wing hue [31,34]. Finally, we observed a negative relationship between host spore load and spore darkness, with heavier infections associated with lighter-colored spores (Figure 7).

## 4. Discussion

Our results provide strong evidence for differences in spore size and shape among clonal genotypes of a common butterfly parasite, suggesting heritable variation in spore morphology. In fact, parasite genotype explained the greatest proportion of variation in both spore area and spore aspect ratio, relative to other host and parasite variables measured here. Spore size also increased with monarch size, and monarchs with larger and darker orange wings tended to have darker colored spores, consistent with the idea that parasite development depends on variation in host quality and resources. Collectively, these results provide support for variation in spore morphology in response to both genetic and environmental factors.

The finding of strong parasite genotype effects on physical traits of *OE* spores is consistent with prior work showing high levels of variation in other phenotypic traits of *OE* clones—namely, virulence and within-host replication. In fact, a consistent finding of prior studies on this host-parasite interaction is genetically-based variation in *OE* virulence as measured by negative effects of parasites on host longevity and body size [18,20]. Virulence in *OE* correlates positively with parasite load, an index of within-host replication, and both replication and virulence further depend on interactions between parasite and host genotypes [22]. Whether differences in spore morphology among the clonal genotypes examined here, correlate with variation in virulence as established by prior work remains to be tested.

A point worth noting is that despite subtle variations, the characteristic shape of *OE* spores is oblong, a shape known to minimize aerodynamic drag. For example, studies of fungal spores showed that the optimal shape for passive dispersal (based on air movements and gravity) is an oval spore with tapered ends [3]. Other work on fungal spores showed that given the same launch speed, larger fungal spores travel farther than smaller ones, because larger and heavier objects have greater momentum [35]. Although prior work on *OE* transmission has not considered aerodynamics to be important [12,36], it seems possible that variation in spore size and shape might affect *OE* transmission. Vertical transmission might be favored by monarchs depositing high numbers of spores immediately surrounding eggs during oviposition, but horizontal transmission could benefit from spores being scattered more broadly and over farther distances, to cover a wide surface area of host plant material. Further work is needed to examine the dispersal of *OE* spores with different morphometric characteristics, and how this dispersal translates to host infection rates.

Monarch characteristics (sex, size and wing coloration) and overall spore load were associated with the size and darkness of *OE* spores, suggesting that host quality and underlying resources can affect the development of physical traits of unicellular parasites. In particular, monarchs with larger wings (a proxy for larger host body size [27]) produced larger and darker parasite spores. If spore size and darkness correlate positively with parasite fitness (for example, by conferring greater spore longevity and resistance to UV light between transmission events), then parasites produced by hosts that secure more resources (as evidenced by larger monarch body size) might themselves have access to greater resources for growth and pigmentation. An alternative explanation for these results is that smaller monarchs might be those that experienced greater spore loads, and spore size might trade off negatively against spore load. However, we found no support for a relationship between *OE* spore size and spore load in the present study, and a follow up analysis showed no support for a negative relationship between spore load and wing area in our data.

Monarch orange pigmentation was associated with spore darkness, such that monarchs with deeper shades of orange (approaching red) produced *OE* spores with deeper shades of amber. One explanation for this finding is that both monarchs and their *OE* parasites use the same raw materials for pigment synthesis, and any variation in the quality or amount of these materials within monarchs can affect both organisms. Pigment synthesis for both monarchs and *OE* spores coincides temporally during the late host pupal stage (2–3 days prior to adult eclosion). In Lepidoptera, the raw materials for pigment synthesis (and synthesis of all other adult tissues) are procured during the larval stage [37], suggesting that variation in larval acquisition of resources could alter parasite characteristics. 

Spore size and pigmentation further depended on host sex in this study, with larger and paler spores from females versus males. These differences could reflect sex differences in immune reactions to *OE* infections [19], or could signal differential competition between the host and parasite for resources for pigment synthesis, since female monarchs tend to have a greater area of black wing pigment than males [28]. That pigmentation synthesis in *OE* spores is resource-limited is further supported by the finding here that *OE* spores tended to be paler in heavy infections (*i.e*., with higher spore loads).

Our findings of relationships between spore physical traits and host characteristics agree with prior studies focused on metazoan parasites [7,8,9,10], and generally support the idea that parasite performance depends on the quality and quantity of finite resources within the host. This point is further emphasized in studies where hosts are deprived of food or stressed in other ways [38,39,40] and parasite performance within these hosts (*i.e*., growth or transmission) is negatively affected.

## 5. Conclusions

This study provides support for heritable variation in spore morphology and points towards a role for host traits in affecting parasite development. Quantifying variation in these traits and establishing their underlying genetic basis is an important first step towards exploring their adaptive significance. Although the current study was not designed to examine the functional significance of *OE* spore traits, directions for future work include examining relationships between morphological traits and parasite fitness (including between-host transmission, spore longevity and resistance to environmental stressors), and conducting a more comprehensive analysis of population divergence in spore traits using a greater number of parasite genotypes. As our results demonstrate, future analyses of trait variation in *OE* and other parasites should take care to control for underlying variation in host characteristics.
